# Socio-economic analysis of the EU citizens’ attitudes toward farmed animal welfare from the 2023 Eurobarometer polling survey

**DOI:** 10.3389/fvets.2025.1505668

**Published:** 2025-03-14

**Authors:** Giorgia Riuzzi, Barbara Contiero, Flaviana Gottardo, Giulio Cozzi, Arzu Peker, Severino Segato

**Affiliations:** ^1^Department of Animal Medicine, Production and Health, University of Padova, Legnaro, Italy; ^2^Department of Animal Health Economics and Management, Ankara University, Ankara, Türkiye

**Keywords:** Eurobarometer, farmed animal welfare, EU survey, cluster analysis, citizen attitude

## Abstract

**Background and methods:**

Europeans’ expectations and opinions regarding the conditions and welfare of farmed animals have evolved continuously. Since 2005, the Eurobarometer (Eb) polling instrument has been used to monitor EU citizens’ attitudes towards farmed animal welfare (FAW). Using the last Eb survey (2023), this study categorized respondents into clusters according to their answers to 12 selected questions on FAW. The ultimate goal was to highlight trends useful to stakeholders and policymakers within the animal food supply chain to design and implement activity planning, progress, and information campaigns.

**Results and discussion:**

As the Eb data came from a stratified multi-stage, random (probability) sample design, the seven clusters sorted through our statistical approach reflected the opinions of the EU population in 2023. These clusters could be further merged into three macro-clusters with two main opposite levels of concern (>80% positive answers) about FAW: concerned about at least 10 questions (74% of the sample); concerned about no more than three questions (6% of the sample); and a third macro-cluster in between concerned about five to seven questions, especially on specific farming practices (20% of the sample). An analysis of the socioeconomic characteristics of the respondents within clusters (gender, age, education, occupation scale, geographical origin, and regular contact with animals) showed that the main discriminating features were gender, level of education, and regular contact with animals; women and well-educated people in regular contact with companion animals were more concerned about FAW overall. The analysis also highlighted divergent responses regarding shopping habits and information searches among the clusters.

## 1 Introduction

Over the last few decades, animal farming supply chains have progressively modified their rearing systems to meet the increasing demand for cheaper food products of animal origin (FPAO) (1). From the sixties to the eighties, to increase production while reducing food prices, agri-food production systems were driven by technological innovation and European agricultural policies^1^ toward farming practices that enhance production performance. In the case of farmed animals, the practices have turned toward genetic selection for productivity, adoption of feeding regimes that are far from natural feeding behavior (chemical composition and physical form of the feeds offered), cage housing systems, high stocking density, automation of husbandry procedures, and use of antibiotics within prophylactic approaches (2). However, such practices have been widely questioned by the public, especially in Europe, regarding environmental sustainability and farmed animal welfare (FAW) (3, 4). That is why European Institutions have actively responded to such requests through legislation and study commissions (5)^2^. Together with complying with the evolving EU legislation on animal welfare (AW), sectors of the animal farming industry, at the same wavelength as the related policymaking authorities and food supply chains, have been trying to steer their farming practices (e.g., feeding, housing, husbandry, transportation) toward welfare-oriented and environmentally sustainable production processes, adopting mostly voluntary certification schemes (6, 7)^3^.

Within this context of evolving individual attitudes (both as citizens who express their concerns overtly, thus affecting public opinion and urging political decision-making, and as consumers), recognizing and investigating the interactions between demographic and socioeconomic traits and individual convictions could offer valuable insights into the various nuances characterizing the opinions of a multifaceted public (8). In fact, over the past 20 years, EU authorities have sought to assess and monitor EU citizens’ opinions and perspectives on AW through the Eurobarometer (Eb). Since 1974, this polling instrument has been used to monitor the social and political attitudes of citizens in EU Member States on a wide range of topics, including EU policies, health, social issues, and the environment, and more^4^. In particular, the 2023 Eb survey provides, by far, the most recent and largest pool of data, offering valuable insight into current EU citizens’ declared attitudes toward AW^5^. However, such resources appear to be neglected by the scientific community, together with survey investigations of the same nature and methods worldwide (8), which have not been using them to their full potential to tailor research according to citizens’ requirements, thus depriving policymakers and other stakeholders of helpful inputs to set the direction of their decisions and carefully design their strategies.

Based on the data collected through the 2023 Eb survey, the present study aimed to:

1. Explore, using cluster analysis, the most current attributes and concerns that identify European citizens’ attitudes toward FAW.
2. Relate the obtained clusters to their main socio-economic features.

This second analysis intended to suggest new approaches for policymakers and the food supply chain to build interactions with citizens to support and improve animal welfare.

## 2 Materials and methods

### 2.1 Data collection and Eurobarometer survey experimental design

The data considered for the present study were obtained from the GESIS Panel data file reporting the full raw individual answers to the Eurobarometer survey conducted between March 2 and 26, 2023 on *Attitudes of Europeans towards Animal Welfare* (9). The survey was conducted at the request of the European Commission by Kantar Public on behalf of Kantar Belgium. It covered the population of residents aged 15 years and older in each of the 27 EU member states, and was conducted as reported in the Technical Specifications of the related report (10). A stratified multi-stage, random (probability) basic sample design was adopted, providing a response sample representing the entire territory of all the countries involved according to the EUROSTAT NUTS II (or equivalent) and the distribution of the resident population of the respective nationalities in terms of metropolitan, urban, and rural areas^6^. The survey was carried out through face-to-face interviews conducted either physically in people’s homes or through remote video interaction (“online face-to-face” or Computer Assisted Video Interviewing, CAVI, only in Czechia, Denmark, Malta, and Finland) in the appropriate national language.

### 2.2 Attitude toward animal welfare: question selection and grouped answers

To evaluate the most current European citizens’ attitude toward FAW, 12 questions (hereby called “question items”) were selected from the 2023 Eurobarometer survey. Table 1 lists the selected question items, possible answers, and answer groupings. When related to living conditions and breeding practices concerning FA (even if not exclusively; e.g., they could also concern companion animals) and FPAO, questions were selected if they investigated consumers’:

- Opinion; QC2, importance of FA protection; QC5, specific farming and breeding practices; and QC12, current offers of animal welfare-friendly food products.
- Desires: QC1, desire for more information on FA condition**s**; QC3, improving FA protection.
- Shopping habits; QC11, on animal welfare-friendly labels on food products.

**Table 1:** Original question items, abbreviation labels of the question items, related possible answers and answer grouping.

Original question items	Question label	Possible answers	Grouped answers
QC1 Would you like to have more information about the conditions in which farmed animals are raised in (COUNTRY)?	More information	Yes, certainly	Yes
		Yes, probably	
		No, probably not	No
		No, certainly not	
		Do not know	Do not know
QC2 In your opinion, how important is it to protect the welfare of farmed animals (e.g., pigs, cattle, poultry, etc.) to ensure that they have decent living conditions?	Importance of farmed animal welfare	Very important	Important
		Somewhat important	
		Not very important	Not important
		Not at all important	
		Do not know	Do not know
QC3 Do you believe that in general the welfare of farmed animals in (COUNTRY) should be better protected than it is now?	Better protection	Yes, certainly	Yes
		Yes, probably	
		No, probably not	No
		No, certainly not	
		Do not know	Do not know
QC5 How important do you consider that each of the following elements is to ensure that farming and breeding practices (both for farmed animals and the breeding of cats and dogs for commercial purposes) meet our ethical responsibilities to animals?			
QC5_1 Banning the cutting of certain body parts of the animals (tails, ears, beaks, testicles, teeth, etc.) unless it’s necessary to protect the safety of workers/farmers (in which case anesthesia will be used)	No mutilation	Very important	Important
		Somewhat important	
		Not very important	Not important
		Not at all important	
		Do not know	Do not know
QC5_2 Ensuring that animals are not kept in individual cages	No individual cages	Very important	Important
		Somewhat important	
		Not very important	Not important
		Not at all important	
		Do not know	Do not know
QC5_3 Ensuring that people who handle the animals have sufficient skills and training	Qualified personnel	Very important	Important
		Somewhat important	
		Not very important	Not important
		Not at all important	
		Do not know	Do not know
QC5_4 Providing farmed animals enough space to be able to move around, lie down and stand up	Enough space	Very important	Important
		Somewhat important	
		Not very important	Not important
		Not at all important	
		Do not know	Do not know
QC5_5 Ensuring that farmed animals have enough food and an adapted environment satisfying their basic needs (e.g., mud, straw, etc., depending on the species)	Food and adapted environment	Very important	Important
		Somewhat important	
		Not very important	Not important
		Not at all important	
		Do not know	Do not know
QC9 Do you think that the travel time for the transport (for ‘commercial purposes’) of live animals within or from the EU should be limited?	Time for transport	Yes, definitely	Yes
		Yes, to some extent	
		No, not really	No
		No, not at all	
		Do not know	Do not know
QC10 In your opinion, how important is it to improve the welfare of animals in slaughterhouses, for example by increasing official controls, including with the use of video cameras?	Welfare at slaughterhouse	Very important	Important
		Somewhat important	
		Not very important	Not important
		Not at all important	
		Do not know	Do not know
QC11 Products sourced from animal welfare-friendly farming systems may carry an identifying label. Do you look for these labels when buying food products?	Welfare-friendly labels	Yes, most of the time	Yes
		Yes, sometimes	
		No, or very rarely	No
		No, never	
		You did not know these labels existed (SPONTANEOUS)	Do not know
		Do not know	
QC12 Do you think there is currently a sufficient choice of animal welfare-friendly food products in shops and supermarkets?	Sufficient welfare-friendly products	Yes, certainly	Yes
		Yes, probably	
		No, probably not	No
		No, certainly not	
		Do not know	Do not know

Two of the selected question items can be considered to cover both consumers’ opinions and knowledge: QC9, on the limitation of time for transport, and QC10, on the need to improve welfare in slaughterhouses. To the selected questions, respondents were allowed to give one among five possible answers: two positive answers, two negative answers, or “Do not know.” The possible answers to each question were grouped into three final categories: positive answer, negative answer, and “Do not know.” Only one question (QC11) provided six answer options, as it included the possibility to spontaneously declare unawareness on the specific item topic, which was embedded in the “Do not know” final grouped category.

### 2.3 Respondents’ socio-economic characteristics

The following socioeconomic variables were selected from the 2023 Eurobarometer questionnaire and taken from the GESIS data file for each respondent: gender, age, highest level of education (according to the International Standard Classification of Education, ISCED 2011), socio-professional category (according to the International Standard Classification of Occupations 2008, ISCO-08), country, and regular contact with animals in daily life. The socio-professional Eurobarometer categories were considered both as they were and as further grouped into “active” and “inactive” population. The Eurobarometer categories for education level and country were further grouped as follows:

- Highest level of education: Primary (pre-primary education, including no education, and primary education); Secondary (lower secondary education, upper secondary education, post-secondary non tertiary, including pre-vocational or vocational education, and education up to ISCED 4 completed abroad); Tertiary (short-cycle tertiary, Bachelor or equivalent, education ISCED 5 and above completed abroad, Master or equivalent, Doctoral or equivalent); “Do not know” (including spontaneous refusal).
- Countries: Central–northwestern Europe (Austria, Belgium, Denmark, Germany, Finland, Ireland, Luxembourg, the Netherlands, Sweden), Eastern Europe (Bulgaria, Czechia, Estonia, Hungary, Latvia, Lithuania, Poland, Romania, and Slovakia), and Southern Europe or Mediterranean areas (Croatia, Cyprus, France, Greece, Italy, Malta, Portugal, Slovenia, Spain).

### 2.4 Statistical cluster analysis

All statistical analyses were performed using SAS 9.4 (SAS Institute, Inc.). The original 26,376 respondents were clustered based on their positive answers to the 12 question items. Clustering was performed using a k-means cluster analysis (PROC HPCLUS), which automatically selected the best k number of clusters. The optimal number of clusters was calculated using the aligned box criterion (ABC) method. Similar to the gap statistics method presented by Tibshirani et al. (11), the ABC method compares the change in the error measure with the change expected under an appropriate reference null distribution. The maximum ABC value corresponds to an adequate number of clusters. The numbering of the final clusters does not vehicle any hierarchical importance and is merely a way to label the final clusters obtained. Clusters were then profiled according to their positive answer patterns and main socioeconomic composition. The percentage of positive answers to each of the original question items for each cluster was calculated, and a heatmap representation was used to display the relationships between the clusters and positive answers to the original items. For the socioeconomic profile, the percentage compositions of each socioeconomic category considered were compared among clusters using a k-proportions test. *Post hoc* pairwise comparisons among proportions were performed using the Marascuilo procedure. Respondents’ age, as a continuous variable, was tested among clusters using an ANOVA approach. This approach allowed us to investigate the relationship between socioeconomic variables and opinions on FAW.

## 3 Results

### 3.1 Descriptive statistics of the 2023 Eurobarometer responding dataset

The distribution of respondents to the Eb survey on EU citizens’ attitudes toward animal welfare (AW) among the 27 Member States and related national response rates are reported in Supplementary Table S1. The average age of the respondents was 51 years, with a slightly higher prevalence of females (53%). Most participants (61%) had secondary education. Respondents with up to bachelor’s, master’s, and doctoral education accounted for 20, 12, and 1%, respectively. Only 55% of the respondents were active with the following professional composition: manual workers (21%), white-collar workers (15%), managers (12%), and self-employed workers (8%). Among the inactive population (45%) there was a high prevalence of retired people (30%), while students (7%), house persons (4%), and unemployed individuals (4%) accounted for a smaller share of the respondents.

### 3.2 Descriptive statistics of the clusters’ attitude toward animal welfare

The iterative k-means clustering procedure based on the ABC criterion resulted in seven clusters representing different levels of positive attitudes toward FAW. As reported in Tables 2, 3, clusters 7 (*n* = 10,587) and 5 (*n* = 8,901) were the most and second-most abundant, respectively, followed by cluster 2 (*n* = 3,556). Clusters 1, 3, 4, and 6 had fewer than 1,000 respondents each. Table 2 presents the percentage of positive answers for each original question item by cluster. In cluster 7, all the original 12 items received more than 80% positive answers, followed by cluster 5, which appeared to be less concerned with the identification of welfare-friendly FPAO and their labels. In contrast, in cluster 1, none of the 12 items received more than 80% of the positive answers, followed by cluster 4, which was concerned only with ensuring qualified personnel, enough space, feed, and suitable environments. In between, clusters 2, 3, and 6 focused their concerns especially on specific farming practices. Across clusters, the item that scored most of the negative answers was “QC1 More information” (5 red cells; red means more than 80% of negative answers), followed by “QC3 Better protection” and “QC11 Welfare-friendly labels” (3 red cells each). Meanwhile the ones concerning respondents the most were “QC5_3 Qualified personnel,” “QC5_4 Enough space,” “QC5_5 Food and adapted environments” (6 green cells each; green means more than 80% of positive answers).

**Table 2:** Cluster distribution on the basis of the positive grouped answers (% in the cells) to the original Eurobarometer question items.

	Cluster (*n*. of respondents; % of total respondents)							
	1 (670; 3)	2 (3,556; 14)	3 (842; 3)	4 (918; 3)	5 (8,901; 34)	6 (902; 3)	7 (10,587; 40)	
Question items	Percentage (%) of positive answers							Eb dataset average
QC1 More information	15	0	11	11	86	0	82	67
QC2 Importance of FAW	20	90	81	16	98	77	97	91
QC3 Better protection	18	81	0	11	95	0	94	84
QC5_1 No mutilation	12	87	69	67	94	80	93	89
QC5_2 No individual cages	13	87	59	62	93	73	93	89
QC5_3 Qualified personnel	18	94	86	82	97	88	96	93
QC5_4 Enough space	15	96	90	82	98	91	96	94
QC5_5 Food and adapted environment	18	97	93	86	98	91	96	94
QC9 Time for transport	24	74	51	15	88	74	90	83
QC10 Welfare at slaughterhouse	20	79	54	5	92	77	93	88
QC11 Welfare-friendly labels	19	0	0	11	54	95	82	60
QC12 Sufficient welfare-friendly products	25	27	81	17	0	70	97	48
Assigned description according to positive grouped answers	Poorly concerned about FAW overall	Concerned especially about specific farming practices	Concerned especially about specific farming practices	Poorly concerned about FAW overall	Highly concerned about FAW overall	Concerned especially about specific farming practices	Highly concerned about FAW overall	

Green-shaded cells: >80% of positive answers, red-shaded cells: >80% of negative answers, white-shaded cells: <80% of both positive and negative answers. The table reports only the percentages of positive answers, not the negative or “Do not know” percentages. FAW, Farmed animal welfare. The numbering of the final clusters does not vehicle any hierarchical importance and is merely a way to label the final clusters obtained.

**Table 3:** Cluster distribution of respondents’ age and socio-economic variables (%).

	Cluster (*n*.; % on total respondents)								
	1 (670; 3)	2 (3,556; 14)	3 (842; 3)	4 (918; 3)	5 (8,901; 34)	6 (902; 3)	7 (10,587; 40)		
Socio-economic variables	Percentages within cluster							Eb dataset average	*p*
Female	47^bc^	50^b^	38^c^	43^c^	56^a^	46^bc^	55^a^	53	<0.001
Age group								51	
≤24	10	8	6	9	9	7	9	9	n.s.
25–39	22	17	16	19	20	17	20	20	n.s.
40–54	27	22	29	24	26	26	26	25	n.s.
≥55	41^b^	53^a^	49^ab^	47^ab^	45^b^	51^a^	45^b^	46	<0.001^1^
Level of education^2^									
Primary	7^ab^	9^a^	5^b^	6^ab^	5^b^	3^b^	5^b^	6	<0.001
Secondary	72^a^	64^b^	62^b^	71^a^	59^b^	59^b^	62^b^	61	<0.001
Tertiary	20^c^	27^bc^	33^ab^	23^cd^	36^a^	38^a^	34^a^	34	<0.001
Socio-professional category									
Active	55	48	56	53	55	54	57	55	
Self-employed	7	6	11	7	8	9	8	8	n.s.
Managers	7	9	11	10	13	14	12	12	n.s.
Other white collars	10	12	12	11	14	13	16	15	n.s.
Manual workers	30^a^	21^bc^	21^bc^	25^ab^	19^c^	18^c^	21^bc^	21	<0.001
Inactive	45	52	44	47	45	46	43	45	
House persons	7	4	3	4	4	4	4	4	n.s.
Unemployed	6	5	3	5	4	3	3	4	n.s.
Retired	26^c^	37^a^	32^abc^	31^abc^	29^bc^	34^ab^	28^bc^	30	<0.001
Students	6	7	5	6	8	6	8	7	n.s.
European region^3^									
Central-Northwestern Europe	23^e^	29^d^	43^ab^	16^e^	34^c^	51^a^	37^b^	35	<0.001
Eastern Europe	59^a^	47^b^	43^b^	64^a^	29^c^	30^c^	31^c^	35	<0.001
Southern Europe (Mediterranean area)	19^cd^	24^c^	14^d^	20^cd^	37^a^	18^d^	32^b^	31	<0.001
Regular contact with animals									
Owned companion animals	23^d^	41^b^	43^ab^	30^bd^	49^a^	37^bc^	47^a^	46	<0.001
Other companion animals	11^c^	13^bc^	13^bc^	12^c^	19^a^	18^ab^	20^a^	18	<0.001
Farming animals	11^a^	5^b^	11^a^	9^a^	5^b^	10^a^	6^b^	6	<0.001
Other animals	7	4	7	6	6	5	5	5	n.s.
Global	43^d^	54^bc^	57^bc^	49^cd^	65^a^	58^b^	66^a^	63	<0.001
Assigned description according to socio-economic variables	Males, younger, secondary education level, manual workers, Eastern European, in contact with farmed animals	Older, primary education level, inactive, retired	Males, in contact with farmed animals	Males, secondary education level, manual workers, Eastern European, in contact with farmed animals	Females, younger, tertiary education level, Southern European, in contact with companion animals	Males, older, tertiary education level, Central-Northwestern European, in contact with farmed animals	Females, younger, tertiary education level, active, Southern European, in contact with companion animals		

Gray-shaded cells indicate significantly (*p* < 0.001) higher values. ^1^ANOVA test. ^2^Primary: pre-primary education (including no education) and primary education; Secondary: Lower secondary education, upper secondary education, post-secondary non-tertiary (including pre-vocational or vocational education), and education up to ISCED 4 completed abroad; Tertiary: Short-cycle tertiary, bachelor’s or equivalent, education ISCED 5 and above completed abroad, master’s or equivalent, doctoral or equivalent. ^3^Central-Northwestern Europe: Austria, Belgium, Denmark, Germany, Finland, Ireland, Luxembourg, the Netherlands, Sweden; Eastern Europe: Bulgaria, Czechia, Estonia, Hungary, Latvia, Lithuania, Poland, Romania, Slovakia; Southern Europe (Mediterranean area): Croatia, Cyprus, France, Greece, Italy, Malta, Portugal, Slovenia, Spain. *p*, *p* value of the k-proportions test; n.s., not significant. Different superscript letters along rows indicate significantly different values. The numbering of the final clusters does not vehicle any hierarchical importance and is merely a way to label the final clusters obtained.

**Figure 1:**
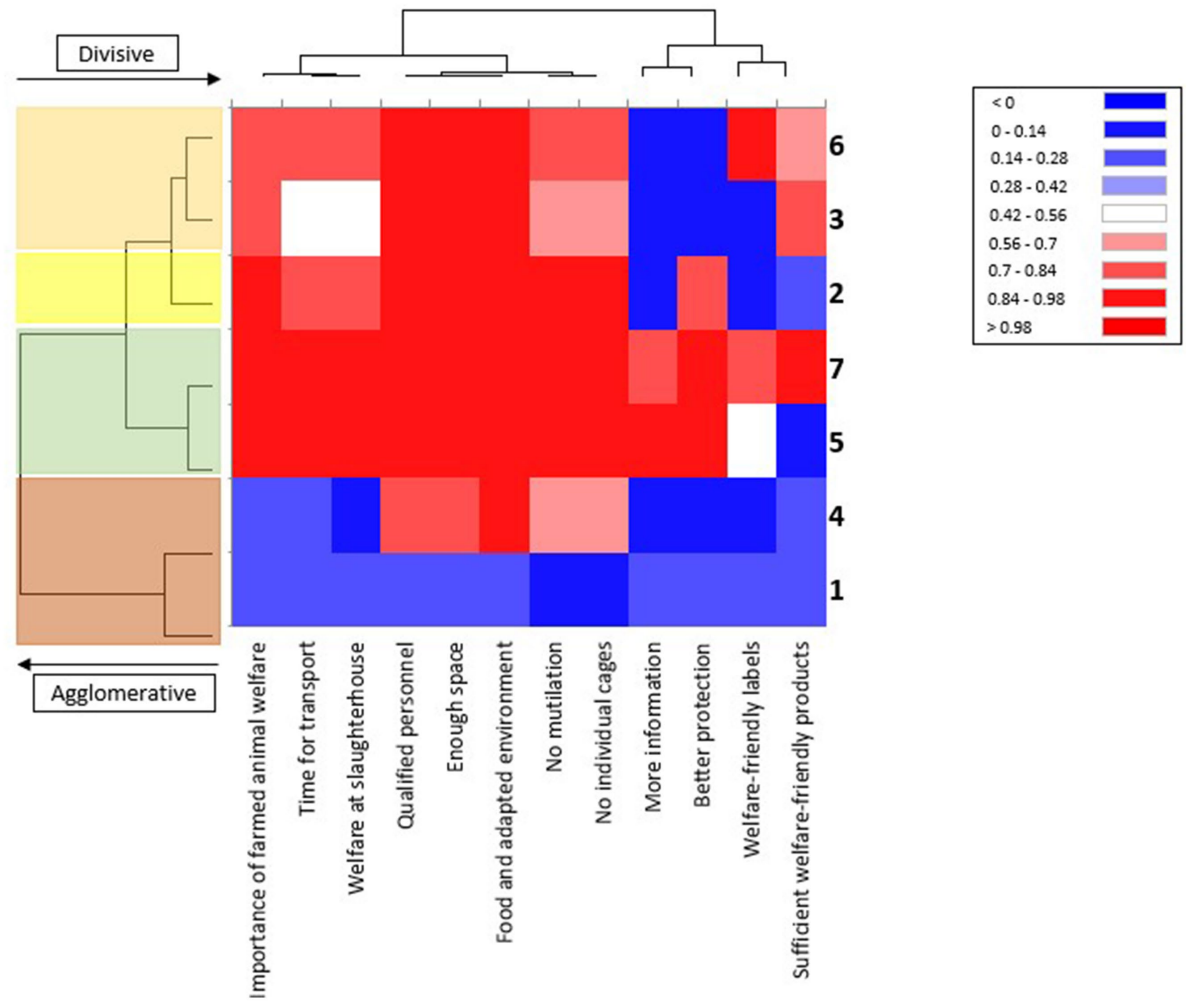
Heat map of the correlations between clusters and positive answers to the 12 question items. In the heatmap, the red (positive) and blue (negative) color scales indicate the degree of correlation. Question items (x axes) and clusters (y axes) are branched together by using a hierarchical cluster analysis. On the left, different colors are used to highlight the same main branched clusters outlined in Figure 2 (from the top to the bottom): clusters 6 and 3 in orange, cluster 2 in yellow, clusters 7 and 5 in green, clusters 1 and 4 in red.

The heat map displayed in Figure 1 shows the correlations between the clusters and positive answers to the 12 question items. It confirmed that clusters 1 and 4 were isolated and similar to one another, with a prevalence of negative answers to most, if not all, items. In addition, the three question items receiving less concerns (i.e., “QC1 More information,” “QC3 Better protection,” “QC11 Welfare-friendly labels”) appeared to be grouped together and with “QC12 Sufficient welfare-friendly products,” which was the item with the most evenly distributed positive and negative answers (49% vs. 41%) and the highest percentage of “Do not know” (10%).

According to the positive answer patterns described above, the clusters were labelled according to the following levels of attitude:

- Clusters 5 and 7: highly concerned about FAW overall.
- Clusters 2, 3, and 6: concerned especially about specific farming practices.
- Clusters 1 and 4: poorly concerned about FAW overall.

**Figure 2:**
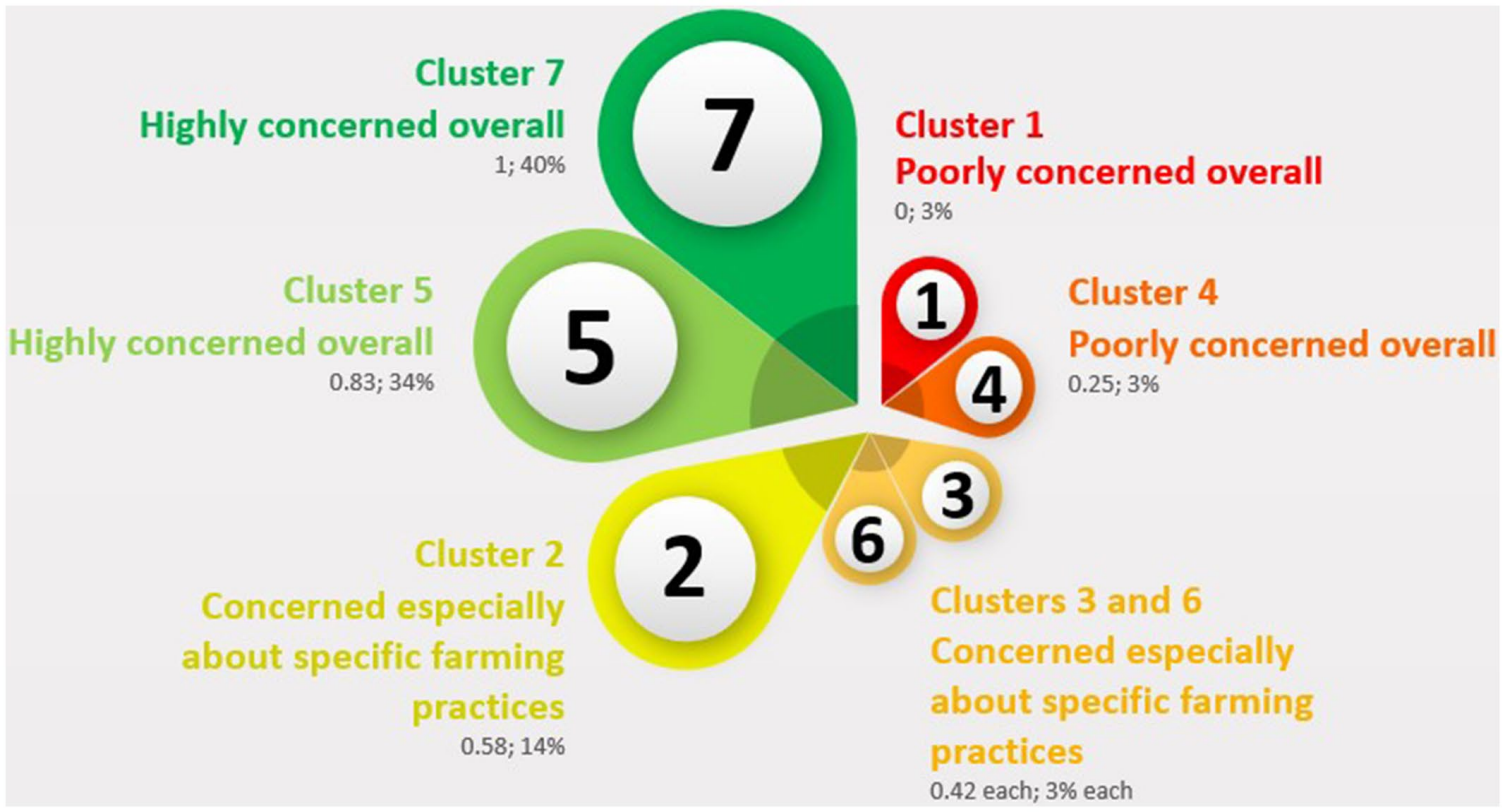
Flower representation of the seven clusters obtained through an iterative k-means cluster procedure on the base of the percentages of the respondents’ positive answers to the original 12 question items. In a clockwise direction, the clusters are presented from the smallest to the biggest, including: their tentatively assigned descriptions according to their percentages of positive answers, their ratio between question items receiving more than 80% of positive answers and the total number of question items, and their percentage of total respondents. Design source: PresentationGO (www.presentationgo.com), reproduced with permission.

Figure 2 displays a flower representation summarizing the main features of the seven clusters based on the percentage of positive answers. The largest petals represent clusters 5 and 7, including most of the respondents (34 and 40%, respectively); while the smallest petals represent clusters 1, 3, 4, and 6 (3% of respondents each); in between, cluster 2 accounted for 14% of the respondents. For each cluster, the figure also reports the ratio of question items that received more than 80% positive answers to the total number of question items.

### 3.3 Descriptive statistics of the clusters’ socio-economic characteristics

The socioeconomic characteristics of the clusters were analyzed for profiling. Table 3 shows the percentage distribution of respondents by socioeconomic variable within the cluster and the statistical differences among the clusters. Clusters 5 and 7 had a significantly higher prevalence of females and younger individuals with a tertiary education level, likely coming from Southern Europe and in regular contact with companion animals. Clusters 1 and 4 appeared to be mainly composed of males, younger individuals with a secondary education level, manual workers likely coming from Eastern Europe, and those in regular contact with farmed animals. In cluster 2, there was a significant overrepresentation of older, inactive, and retired people with a primary education level, whereas cluster 6 appeared to be composed of a significantly higher percentage of males, older individuals with a tertiary education level, likely from Central-Northwestern Europe, and in regular contact with farmed animals. Finally, cluster 3 was characterized only by a higher prevalence of males in regular contact with farmed animals.

## 4 Discussion

The cluster analysis implemented in the present study identified seven clusters, more than those of another recent work by de Boer and Aiking (12), which was also conducted at the European level and based on a 2022 Eb survey on food and safety, where five clusters were obtained. However, if described according to the percentages of positive answers (>80%, Table 2), our seven clusters can be grouped into three larger macro-clusters characterized by two opposing attitudes for the extreme groups (clusters 1 and 4 vs. clusters 5 and 7) and, in the middle, a group whose concerns focused especially on specific farming practices (clusters 2, 3, and 6). Nonetheless, considering the weight of such clusters (Figure 2), the ones that were highly concerned about farmed animal welfare (FAW) overall include the vast majority of respondents (74%), while the poorly concerned ones represented only a small minority (6%).

The total number of 2023 Eb respondents accounted for 0.007% of the total European population over 15 years old in the 27 Member States, which was higher than the population sampled in other investigations carried out in the last few decades on European citizens’ opinions on animal welfare (AW), conducted only in a selection of representative European countries (13–16). This confirms that the Eurobarometer provides by far the most comprehensive datasets within the European context. However, very few other peer-reviewed articles resulted when searched through scientific literature databases, such as Scopus, and using the following string: “Eurobarometer AND animal AND welfare.” As of 2005, when the first Eb survey on FAW was published, nine results were found. Of these, only two papers were relevant and comparable in terms of the investigated topics, data, and work methodologies.

As a result of a stratified multi-stage, random (probability) sample design in terms of overall socioeconomic variables, and if compared with Eurostat data, the final respondent sample was representative of the socioeconomic composition of the actual 15-and-over population in 2023. In fact, a stratified multistage sampling appears to be the most suitable choice for large-scale monitoring surveys that aim at assessing status, change and trends of one or more parameters within a highly geographically diverse group, such as the 15-and-over European population; being both practical and effective and cost and time saving (17, 18). As a multistage random approach, where nested or hierarchical structure of the members within the population is taken into account, and then arranged in clusters that will be randomly sampled at each stage, it ensured a representative final sample of the original population (16, 19). Finally, as a stratified sampling designed according to the EUROSTAT NUTS II (or equivalent) and the DEGURBA Urban Rural classification, it ensured representative coverage based on the entire national territory (10, 17, 19). In light of the great representativeness of the final sample of respondents to the 2023 Eurobarometer survey, the clusters produced through our statistical approach are a representative clusterization of both the attitudes toward FAW of the actual EU population and of its socio-economic characteristics.

The majority (74%) of the respondents, represented by clusters 5 and 7, who were highly concerned about (almost) all the investigated question items about FAW, could also be profiled as being mainly composed of females, younger individuals, with a tertiary education level, likely from Southern Europe, and in regular contact with companion animals. Indeed, such clusters included most respondents that, in their daily lives, were in regular contact with animals overall, especially companion animals, as opposed to clusters 1 and 4 that included most of those who were in contact with farmed animals (Table 3). It should be noted that a source of bias influencing respondents’ answers could have arisen from the formulation of QC5 (Table 1). Since it explicitly specified that the practices listed in the question could have been applied both to *“farmed animals and the breeding of cats and dogs for commercial purposes,”* it might have instilled a deeper feeling of sympathy in the respondents, coming from the involvement of companion animals (20). From our findings, the answers to both “QC1 More information” and “QC3 Better protection” also confirmed the trend described above, with a majority of respondents calling for a better protection of FAW in their own countries (88%; i.e., clusters 2, 5, and 7) and 74% of individuals declaring a desire for more information on FAW (i.e., clusters 5 and 7). This shows once more that this portion of respondents is more skeptical about the fact that animals are kept in good condition, and therefore, ask for further information and improvements in FAW.

Such higher levels of concern about FAW topics among women, younger citizens, and those who have spent more time in education, both in Europe and across the world, have already been reported on many occasions (3, 21–24). The importance placed on AW appears to decrease with age, as older individuals are more readily accepting current welfare standards and have more production-related views of animals (25), which is in line with the moderate attitude held by older respondents in clusters 2 and 6. However, although there have been studies reporting lower levels of knowledge about actual farming, transportation, and slaughter conditions and practices (26, 27), from our findings we cannot establish neither what is the factual knowledge that Eb respondents had on AW or how such knowledge was acquired by the investigated sample of EU citizens. In contrast to previous Eb surveys (2007 and 2016), in 2023, no questions were asked to the interviewed citizens about their knowledge of the topic or the source of information they relied on (28, 29). In fact, although without implying that attitudes must only be based on factual knowledge nor that they would be necessarily influenced by it, such data would provide valuable inputs for designing information strategies by policy makers, in the first place, and by the food supply chain. Despite a lack of concrete knowledge, consumers were reported to share the impression that the living conditions of farmed animals are far from optimal, especially in conventional production systems (30, 31). This impression, leading to the inevitable conclusion that animals suffer despite all the advances made in animal farming, once again appeared to be typical of women, younger, and well-educated individuals in professional positions, who, therefore, tend to hold on to their negative views of modern farming. Studies have highlighted that these categories of people are more exposed to vague and general information that do not come from thoughtful, accurate, and contextual sources. As a consequence, it is harder for them to realize that farming can be completed also via systems that are both sustainable and animal friendly, and that such systems may differ significantly from the narrative they are exposed to (31, 32). The results of the present study showed that even if it was a small group, likely mirroring an equally small share of farmers and agricultural workers^7^, those in regular contact with farmed animals held a less critical position toward modern farming systems, echoing other studies that have shown divergent perceptions between ordinary citizens and livestock producers or other animal scientists (e.g., veterinarians) (33–35). It must be noted that the attitudes of such a smaller share of respondents (clusters 1 and 4) may be driven by being too accustomed to the reality of breeding and farming practices, or by their likely more pragmatic view of animals’ roles, which makes them readily accept current welfare standards. Furthermore, as clusters 1 and 4 are mainly made up of manual workers and individuals with a secondary level of education, they might have less disposable income allowing them to purchase food products that may be perceived as more expensive (i.e., animal welfare-friendly food products) (1). Indeed, by looking at their answers to QC11, it seems that they were less likely to search for such products, especially when compared to cluster 7, where 82% of the respondents declared that they were looking for animal welfare-friendly products.

Finally, the 2023 Eb survey did not contain any questions investigating the respondents’ lifestyles in terms of diet and religious beliefs, which could greatly affect their approach to FAW (8). Among the majority of highly concerned respondents just described and the smallest group (6%) of citizens that appeared to be poorly involved in the topic, there was a share of respondents (21%) who were concerned mainly about specific FAW aspects regarding farm facilities and rearing systems, namely qualified personnel, enough space and food, adapted environments, mutilations, and the use of individual cages. In fact, these specific farming practices appeared to be a concern for almost all surveyed individuals (97%; all but cluster 1; Table 2 and Figure 1). This is consistent with the findings of Pejman et al. (15) who, still in 2019, reported that AW was most frequently put in relation with the general concepts of “natural outdoor conditions,” “clean and healthy housing environments,” and “good feeding.” According to recent studies, the public seems to be more aware of and sensitive to aspects and practices that have been put under the spotlight of animal protection associations through information campaigns^8^ (1, 24, 36). Furthermore, European studies have reported that animal farming systems are perceived as less animal friendly when there is a lack of free movement, too little space per animal, and non-transparent locked systems, especially for pigs and poultry (30, 37–39). These are the more common farming conditions in Southern Europe, which could partly explain the significantly higher proportion of respondents in clusters 5 and 7. However, it should be noted that this is in contrast to the biosecurity protocols required in highly specialized rearing systems and the necessary adaptations of the rearing techniques, such as grazing practices or outdoor living, to the specific adverse climate conditions (heat stress, rain, and wind) and breed characteristics that make it necessary to protect the animals (40). On the other hand, transport and slaughter conditions still appear to be underrated AW issues; perhaps because they are perceived as short-term issues compared to on-farm living conditions that persist during the entire animal’s lifetime or matter with few obvious alternatives. Furthermore, the most recent information campaigns run by animal protection associations have focused more on animals’ on-farm life stages, rather than on transport and slaughter, to raise awareness of topics such as individual cages and mutilation^9^.

If looking at the present results from a geographical perspective, it emerged that respondents from Eastern Europe were less concerned about FAW overall, as opposed to other European countries, many of which have been exposed and sensitized to FAW issues since the seventies (5). This might be of valuable support to European policymakers and authorities, as it further suggests that considerations and decisions should be tailored to both the targeted socioeconomic groups and specific geographical zone (8).

The answers to QC11 and QC12 highlighted whether the respondents looked for labels identifying welfare-friendly food products, and whether they thought there was a sufficient choice of such foods in shops and supermarkets. The analysis of the answers showed a certain level of disagreement among clusters and within previously unanimous socioeconomic groups. A total of 43% of the 2023 Eb respondents looked for welfare-friendly labels, mainly female, younger, and well-educated individuals, likely from Southern Europe, and in regular contact with companion animals. A decreasing percentage, if compared to 2016, when the same question was paused and 52% of the respondents answered positively (28). It is possible that, as consumers, EU citizens do not want any further labels on FPAO, and it is likely that such products respect animal welfare as a necessary prerequisite. Otherwise, a dissociation might be observed between what respondents declared through their answers and what they actually looked for when shopping. Alonso et al. (3) found that although FAW has been viewed as an increasingly important attribute of food quality and safety, EU consumers still prioritize their own human safety and individual benefits over societal or animal benefits *per se*, using AW more as an indirect indicator for human health. Moreover, while in our previous results, the young, female, and well-educated socioeconomic group represented by clusters 5 and 7 was always in agreement on every question item, it split when it came to QC11 and QC12, with half of them stating that they look for welfare-friendly labels and think there are enough welfare-friendly foods (40%; cluster 7) and the other half not searching for nor thinking there are enough of such products (34%; cluster 5). Furthermore, the evenly distributed overall positive and negative answers (49% vs. 41%, respectively) and the highest prevalence of “Do not know” (10%) to “QC12 Sufficient welfare-friendly products” testified to the lack of awareness surrounding these products, which seemed to be highly disregarded since 20% of the respondents openly declared they do not search for labels identifying them (clusters 2, 3, and 4). For this reason, it is crucial to understand the drivers of the respondents’ shopping habits and have more information about their dietary regimes (omnivores, vegetarians, vegans), whether they place importance on food price and are responsible for shopping, and who they trust for certifying products’ origins. In general, even if perhaps ambitious, knowing the respondents’ ethics in relation to animals and nature would be extremely helpful in driving conclusions, especially given the increasing divergence between science-based welfare assessments (i.e., ethological issues, physiological parameters, and health status) and ethical assessments involving ethical, socioeconomic, cultural, and religious aspects. Unfortunately, these data were not collected through the 2023 Eurobarometer survey. Furthermore, a discrepancy between self-reported public concerns about FAW and shopping habits has been widely reported and investigated, along with consumer dissociation between live animals and the food they produce (3, 41, 42). For example, although EU citizens appeared increasingly worried about FAW, they were not willing to cover the costs of FAW improvement. This phenomenon was confirmed by the results of the Eb surveys conducted over the years, where a slightly increasing share of more than a third of the respondents declared that they were not willing to pay more for welfare-friendly products (34, 35, and 37% in 2005, 2016, and 2023, respectively) (10, 28, 43). Finally, recent literature has discussed that although consumers welcome additional labeling on production quality overall, they also accept simple labeling better (44). This calls for accurate but accessible information provision to fill the knowledge gap and distance between actual and perceived reality, thus boosting confidence in food chain stakeholders, as well as for simple and clear labeling schemes to promote informed purchasing decision-making as competent consumers. Implementation strategies for accessible animal welfare labeling are still one of the core topics for agricultural policy at the EU level (45).

## 5 Conclusion

Through this study, based on the most recent Eb survey (2023) on EU citizens’ attitudes toward animal welfare, the Eurobarometer appeared to provide a comprehensive dataset capable of capturing the opinions of the composite demographic and socioeconomic categories of the 15-and-over EU population.

As one of the very few research attempts to utilize the Eurobarometer survey, in our reappraisal of the survey data, a cluster analysis allowed the identification, quantification, and further examination of multiple intertwined strands characterizing EU citizens’ opinions in relation to the socioeconomic category to which individuals belong. The seven resulting respondent clusters could be further merged into three macro-clusters with two oppositely high (74% of the sample size) and poor (6%) levels of concern for animal welfare, and a third macro-cluster in between, concerned especially about specific farming practices (20% of the sample). However, a certain level of disagreement among clusters and within socioeconomic groups emerged in terms of shopping habits, with only a minority of the respondents actively looking for welfare-friendly labels on the FPAO. It would be useful to investigate what the actual EU citizens’ attitudes and desires are on label schemes.

In terms of socioeconomic composition, such aggregated clusters seemed to be driven mainly by gender, level of education, and regular contact with animals, with women and well-educated people in regular contact with companion animals being more concerned about farmed animal welfare overall. Above all, it also appears that the implementation of animal welfare strategies and assessment protocols, tailored to both the socioeconomic group and the specific European zone, needs to be better combined with effective and simple communication to the wider public and aiming at helping citizens orient their shopping actions.

Furthermore, the outcomes of the cluster profiling could contribute to informing policymakers and stakeholders within the animal food supply chain to promote more welfare-friendly scenarios according to the concerns of different socio-economic groups. Detailed knowledge of the different social layers’ opinions and attitudes might help establish better conflictless ways to make the two ends of the food supply chain (i.e., producers and consumers) approach each other.

## Data Availability

The Eurobarometer datasets presented and discussed in this study are openly available at: https://www.gesis.org/en/home (accessed on October 2, 2024). Specifically, the datasets coming from the 2023 Eurobarometer survey is available at: https://search.gesis.org/research_data/ZA7953?doi=10.4232/1.14081 (accessed on October 2, 2024). The Eurobarometer reports and questionnaires are available at: https://europa.eu/eurobarometer/surveys/browse/all (accessed on October 2, 2024).

## Footnotes

^1^Timeline - History of the CAP. https://www.consilium.europa.eu/en/policies/cap-introduction/timeline-history-of-cap/ [Accessed October 2, 2024].

^2^EU animal welfare legislation. https://food.ec.europa.eu/animals/animal-welfare/eu-animal-welfare-legislation_en# [Accessed October 2, 2024]. Animal welfare | EFSA. https://www.efsa.europa.eu/en/topics/topic/animal-welfare [Accessed October 2, 2024].

^3^ClassyFarm. https://www.classyfarm.it/index.php/en/ [Accessed October 2, 2024].

^4^50 years of listening to Europeans’ opinions. Celebrating 50 years of Eurobarometer, the European Union’s public opinion service. https://ec.europa.eu/stories/50-years-eurobarometer/ [Accessed October 2, 2024].

^5^What is the Eurobarometer. https://europa.eu/eurobarometer/about/eurobarometer [Accessed October 2, 2024].

^6^Urban Rural classification based on DEGURBA. https://ec.europa.eu/eurostat/web/degree-of-urbanisation/information-data [Accessed October 2, 2024].

^7^Farmers and the agricultural labor force – statistics. https://ec.europa.eu/eurostat/statistics-explained/index.php?title=Farmers_and_the_agricultural_labour_force_-_statistics [Accessed October 2, 2024].

^8^https://food.ec.europa.eu/animals/animal-welfare/eci/eci-end-cage-age_en. https://citizens-initiative.europa.eu/initiatives/details/2018/000004/end-cage-age_en. https://www.endthecageage.eu/en/. https://www.four-paws.org/campaigns-topics/campaigns/end-the-cage-age. [All accessed October 2, 2024].

^9^Eurogroup For Animals – Campaigns. https://www.eurogroupforanimals.org/what-we-do/campaigns [Accessed October 2, 2024].
