# Supplementary material for: Socio-economic analysis of the EU citizens’ attitudes toward farmed animal welfare from the 2023 Eurobarometer polling survey
Source: Front Vet Sci. 2025 Mar 14;12:1505668. doi: 10.3389/fvets.2025.1505668 (PMC11951868; doi:10.3389/fvets.2025.1505668)
Supplement: Supplementary file 1 [file Table_1.docx]

Supplementary Material

Table S1. Distribution of the 26,376 15-and-over respondents to the 2023 Eurobarometer survey among the 27 Member States and the related national response rates

| Country | Country ID | Respondents (n.) | Respondents  (% of total respondents) | Response rate  (%) |
| --- | --- | --- | --- | --- |
| Central-Northwestern Europe |  |  |  |  |
| Austria | AT | 1,011 | 3.83 | 41.9 |
| Belgium | BE | 1,039 | 3.94 | 43.9 |
| Germany | DE | 1,531 | 5.80 | 22.4 |
| Denmark | DK | 1,006 | 3.81 | 38.2 |
| Finland | FI | 1,011 | 3.83 | 24.4 |
| Ireland | IE | 1,005 | 3.81 | 36.2 |
| Luxembourg | LU | 507 | 1.92 | 26.0 |
| Netherlands | NL | 1,001 | 3.80 | 67.7 |
| Sweden | SE | 1,022 | 3.87 | 62.4 |
|  |  |  | Total: 34.62 | Average: 40.3 |
| Eastern Europe |  |  |  |  |
| Bulgaria | BG | 1,024 | 3.88 | 46.5 |
| Czechia | CZ | 1,014 | 3.84 | 49.0 |
| Estonia | EE | 1,008 | 3.82 | 45.7 |
| Hungary | HU | 1,029 | 3.90 | 59.5 |
| Lithuania | LT | 1,011 | 3.83 | 42.4 |
| Latvia | LV | 1,000 | 3.79 | 44.4 |
| Poland | PL | 1,004 | 3.81 | 47.3 |
| Romania | RO | 1,044 | 3.96 | 53.8 |
| Slovakia | SK | 1,003 | 3.80 | 54.5 |
|  |  |  | Total: 34.63 | Average: 49.2 |
| Southern Europe (Mediterranean area) |  |  |  |  |
| Rep. of Cyprus | CY | 504 | 1.91 | 47.7 |
| Greece | EL | 1,006 | 3.81 | 30.2 |
| Spain | ES | 1,013 | 3.84 | 33.4 |
| France | FR | 1,001 | 3.80 | 36.7 |
| Croatia | HR | 1,020 | 3.87 | 40.6 |
| Italy | IT | 1,027 | 3.89 | 23.0 |
| Malta | MT | 516 | 1.96 | 80.1 |
| Portugal | PT | 1,002 | 3.80 | 44.4 |
| Slovenia | SI | 1,017 | 3.86 | 39.5 |
|  |  |  | Total: 30.75 | Average: 41.7 |

# The national response rates were calculated by Kantar Public by dividing the total number of complete interviews with the number of all the addresses visited, apart from those that were not eligible but including those where eligibility was unknown.
